# Target Trial Emulation or Target Trial Approximation?

**DOI:** 10.1002/pds.70485

**Published:** 2026-10-17

**Authors:** Priya Vart

**Affiliations:** ^1^ Department of Clinical Pharmacy and Pharmacology University Medical Centre Groningen, University of Groningen Groningen the Netherlands; ^2^ Groningen Kolff Centre for Kidney Research University Medical Centre Groningen, University of Groningen Groningen the Netherlands

**Keywords:** causal inference, observational studies, randomized controlled trial, real‐world data, target trial approximation, target trial emulation

## Abstract

Target trial emulation has contributed to improvements in the design and interpretation of observational studies aimed at causal inference. By encouraging researchers to think explicitly about the design of a hypothetical randomized trial, it has helped identify and address avoidable design flaws. However, the framework cannot overcome biases and limitations inherent in observational data. Despite the aim of emulating a randomized trial, observational data cannot reproduce random treatment assignment or blinding, and may not necessarily provide greater generalizability than a randomized trial. Thus, the term “emulation” may imply a degree of similarity to a randomized trial that observational data cannot realistically achieve and deserves critical reconsideration to better reflect the limitations of observational data.

## Key Points

- Target trial emulation has improved the design and validity of observational research aimed at supporting causal conclusion.
- It provides a structured framework for addressing avoidable design flaws.
- However, observational studies remain vulnerable to biases inherent in the underlying data.
- The framework cannot fully reproduce the conditions of a randomized target trial necessary for causal inference.
- The term “emulation” deserves reconsideration to encourage more critical interpretation of findings and better reflect what observational data can realistically deliver.

## Plain Language Summary

Target trial emulation has helped improve how observational studies are designed and interpreted. However, observational data still have important limitations and biases that cannot be fully overcome. The term “emulation” may suggest that observational studies can closely reproduce randomized trials, when in practice they can only approximate them

## 1 Introduction

Over the past decade, target trial emulation has emerged as one of the key conceptual frameworks in observational epidemiology [1]. At its core, the framework encourages investigators to articulate a causal question by first specifying the randomized controlled trial (RCT) they would ideally conduct, and then structuring the observational analysis to mirror that trial as closely as the data allow [2]. By requiring investigators to explicitly define eligibility criteria, treatment strategies, assignment mechanisms, follow‐up, outcomes, estimands, and analytic approaches as though designing a randomized trial, the framework has introduced a level of methodological discipline that was frequently absent in earlier observational research. In doing so, it has contributed to the identification and correction of some of the most persistent design flaws in the literature, particularly those arising from misalignment in the timing of exposure, eligibility criteria, and follow‐up [3].

This contribution must not be understated. Many longstanding discrepancies between observational studies and RCTs can be attributed to avoidable design flaws. Studies that compare prevalent users with non‐users, start follow‐up too early or too late relative to treatment eligibility, or define outcomes without aligning them to treatment initiation are prone to immortal time bias, lead‐time bias, and inappropriate comparisons between patients at different stages of disease. By formulating a pre‐defined “time zero” and attempts to ensure that follow‐up begin at a point that aligns eligibility and treatment initiation, target trial thinking has helped correct misleading inferences across several clinical domains. A well‐known illustration has been in the case of postmenopausal hormone therapy where early observational studies reported lower rates of coronary heart disease among women using combined estrogen–progestin therapy [4, 5]. However, these studies initiated follow‐up at cohort entry rather than at treatment initiation, leading to biased comparisons. In contrast, randomized trials later demonstrated that combined estrogen–progestin therapy increased the risk of coronary heart disease [6]. When observational data were subsequently analyzed using a target trial emulation approach that aligned eligibility, treatment initiation, and follow‐up, the findings were consistent with that of randomized trials [7]. More broadly, clearly specifying the elements of a hypothetical trial has improved the identification of appropriate confounders and informed analytic choices. For example, when emulating trials comparing living versus deceased kidney donation, the focus appropriately shifted to recipient characteristics rather than donor factors [8]. In these ways, the framework has raised the methodological standard for observational research aiming to support causal conclusions.

## 2 Distinction Between Emulation and Causal Reproduction

As the application of target trial emulation has expanded, unfortunately, the distinction between designing an observational study informed by a hypothetical trial and reproducing the causal properties of a RCT has become less clear. Applications of target trial emulation have often focused on investigating the effectiveness of therapies in populations that are understudied in RCTs (e.g., statin in older adults, sodium–glucose cotransporter 2 (SGLT2) inhibitors in patients with kidney failure) or on comparing two interventions in situations where conducting an RCT would be challenging in practice [9, 10, 11]. In this context, the language of “emulation” is often taken to imply equivalence with RCTs, which warrants careful consideration. The distinction between implying causality by attempting to mimic an RCT using observational data and establishing causality through an RCT itself is important because terminology shapes expectations, and expectations influence interpretation. Because target trial emulation explicitly aims to “emulate” a hypothetical RCT, it must be assessed against the core features that give RCTs their strength for causal inference.

### 2.1 Emulating Randomization

One of the key strengths of RCTs is that treatment assignment is randomized and thus independent of patient prognosis. It controls for unmeasured and measured confounding. Can a target trial emulation emulate randomization and control for both unmeasured and measured confounding? In observational settings, treatment initiation reflects clinical decision‐making embedded in real‐life healthcare setting where decisions are influenced not only by measured variables such as age, comorbidity, or laboratory values, but also by factors that are often absent from most datasets: frailty, functional status, symptom burden, cognitive impairment, patient preferences, clinician risk tolerance, and access to care [12, 13, 14, 15]. For instance, the prescription of statins in older adults depends not only on patients' cholesterol levels but also on their level of frailty and life expectancy from the physician's perspective [16, 17], as well as cognition and treatment goals as older adults may prioritize health related quality of life over lowering the risk of cardiovascular disease. These data are rarely, if ever, recorded in administrative databases. Importantly, these illustrate the challenges in controlling for known unmeasured confounders and highlight the unavoidable inability to control for unknown unmeasured confounders. Furthermore, for the same research question, variables such as smoking and body mass index are relevant to control for when comparing statin initiators and non‐initiators and are nearly always accounted for studies investigating such question. However, it is not uncommon knowledge that the quality of such data recorded in administrative databases is suboptimal. In an analysis of smoking status related data from electronic health registration at the NewYork‐Presbyterian Hospital/Columbia University Medical Center, 36.4% were documented as “unknown” and 54.5% of patients had implausible changes (e.g., changes from “current smoker” to “never smoker”) [18]. Similarly, body mass index is recorded less frequently and usually for individuals with a higher burden of comorbidities [19]. Adjustment methods, however sophisticated, cannot fully compensate for the absence of information. Propensity scores, inverse probability weights, and marginal structural models can balance what is measured, but they cannot balance what is unknown or poorly captured. Of note, the performance of propensity scores and inverse probability weights explicitly depends on “no unmeasured confounders” assumption [20, 21]. Although proxy adjustments through comparison of similar indications may improve control of confounding compared with user versus non‐user design, residual confounding may still remain. For example, when comparing DPP‐4 inhibitors with SGLT2 inhibitors, patients initiating these treatments often differ in disease severity, cardiovascular and renal risk, frailty, and comorbidity [22, 23, 24]. Thus, it will not be incorrect to acknowledge that target trail emulation of routinely available observational data clearly cannot control for unmeasured confounding and has important limitations even in controlling for measured confounding. In fact, the lack of randomization in the observational data have been acknowledged as a fundamental challenge for the emulation [25].

### 2.2 Emulating Blinding

Can target trial emulation emulate blinding? While blinding may be less of a concern in outcomes like mortality, it remains important for outcomes that are subjective or susceptible to differential assessment. Consider an observational study evaluating whether initiation of an SGLT2 inhibitor, compared with a renin–angiotensin system inhibitor, is associated with kidney disease progression in patients with advanced chronic kidney disease (eGFR < 25 mL/min/1.73 m^2^)—a population subgroup not sufficiently represented in major SGLT2 inhibitor trials. One of the most commonly investigated outcome in these studies is a composite kidney endpoint that included a specified percentage decline (e.g., 50%) in estimated glomerular filtration rate (eGFR) along with other kidney related clinical outcomes (e.g., dialysis initiation). In routine clinical care, knowledge of provided treatment may influence the frequency of serum creatinine testing and eGFR estimation, particularly in SGLT2 inhibitor groups, given the well‐recognized early and acute decline in eGFR after treatment initiation. This differential monitoring may, in turn, affect event detection between groups and increase the likelihood of observing a prespecified eGFR decline in the SGLT2 inhibitor group compared with the renin–angiotensin system inhibitor group, ultimately biasing the estimated treatment effect. Similarly, a study investigating the effect of statin use on dementia (outcome for which RCTs are seldom powered) using observational data may be prone to bias if patients initiating statin therapy are more likely to seek medical attention for cognitive symptoms because of heightened awareness of potential adverse effects, and are therefore more likely to receive a diagnosis of dementia. Indeed, in such a study, the likelihood of being diagnosed with dementia was 46% higher among statin initiators compared with non‐initiators [26]. In the same vein, imaging‐based outcomes (e.g., cardiac remodeling, tumor progression), patient‐reported and functional outcomes are particularly vulnerable. Measures of pain, fatigue, mood, or physical function are influenced by expectations and reporting behavior, which differ systematically when treatment assignment is known [2]. Target trial emulation cannot ensure blinding and cannot fully account for the downstream consequences of unblinded outcome assessment.

### 2.3 Alignment of Time‐Zero

Target trial–informed designs emphasize alignment of eligibility criteria, treatment initiation, and start of follow‐up [27]. However, in routine care, treatment initiation frequently occurs after study entry, and the delay between eligibility and treatment may vary across patients, complicating alignment of time zero and introducing risk of immortal time bias [28]. In older adults, for example, statin initiation frequently occurs at variable times and is often delayed because of polypharmacy, the need to modify or discontinue interacting therapies, evolving comorbidity, or intercurrent clinical events [14]. Consequently, there is often no single, well‐defined point at which both initiators and non‐initiators are simultaneously eligible and under equivalent clinical consideration for treatment, which is a prerequisite for valid comparison. Of note, individuals classified as non‐initiators may include patients in whom treatment is deferred indefinitely due to frailty or competing care priorities, introducing confounding by contraindication [29]. Nonetheless, anchoring follow‐up to treatment initiation creates structurally different time zeros for initiators and non‐initiators, which undermines comparability. Although techniques such as sequential target trial emulation with cloning and inverse probability weighting can be used to impose a common time zero, the resulting estimates depend on the assumption of non‐informative artificial censoring (conditional on measured time‐varying covariates), which may be difficult to achieve and justify clinically in older patients with complex, dynamic care trajectories. For instance, older patients often experience events like falls, acute infections, or cognitive decline that lead to treatment changes. If these treatment changes or health declines determine the artificial censoring, the assumption that censoring is independent of the outcome (non‐informative), even when adjusted for measured time‐varying confounders, may not be met due to data limitations and lack of randomization.

### 2.4 External Validity

Does target trial emulation offer greater external validity? The assumption that real‐world data confer greater generalizability than RCTs is widespread. However, administrative data are often single center and by design captures information on those who actually receives treatment, not who should. Because prescribing reflects both undertreatment of the intended use population and overtreatment of those outside it [30, 31, 32], the resulting study population can be systematically skewed in both directions. The eligibility criteria of most emulated trials are then shaped to fit these data, reinforcing rather than correcting the mismatch. Non‐clinical determinants of prescribing behavior (e.g., social, commercial, and political) further add to the discrepancy between those who get the medication and those most likely to benefit [30, 33]. For instance, in a study examining how different doses of buprenorphine affect withdrawal symptoms in patients being treated for opioid use disorder in Veterans Affairs population, only captured data from fewer than one‐fifth of the eligible Veterans Affairs population, leaving out a majority who likely differ in age, race, socioeconomic status, and comorbidities such as psychiatric disorders and chronic pain. Moreover, the findings may not extend to the far larger population of Americans outside the Veterans Affairs system who likely also differ demographically and clinically from Veterans Affairs population [30]. Effect estimates from such emulations are therefore of uncertain applicability to the intended use population, regardless of methodological robustness.

## 3 Emulation or Not

Taken together, the target trial emulation framework is most effective when bias primarily arises from design flaws that can be identified and corrected. It is less reliable for robust causal interpretations when bias arises from structural limitations inherent in observational data which are unfortunately far too common in routinely collected health care data. The lack of optimal concordance between findings from emulated trial data and RCTs was evident even in emulations of highly selected trials, where inclusion was based on the feasibility of achieving satisfactory emulation. In such a recent study, discordance in findings between emulated analysis and target RCT was substantial where 28% of comparisons failed to agree on statistical significance and 34% failed to achieve estimate agreement [34]. When trial design could not be closely replicated (50% of the included RCTs), agreement dropped sharply, with only 50% statistical significance agreement and a correlation coefficient of 0.53 between conclusions from RCT and target trial emulation. This finding demonstrates that, even under highly controlled and selective conditions, close emulation is frequently unattainable. Moreover, when such emulation cannot be achieved, the likelihood of drawing valid inferences about an intervention's effect is no better than a flip of a coin. Partial concordance should not be surprising as observational and experimental studies address related, but not identical questions and operate under fundamentally different data‐generating mechanisms. Thus, the lack of complete agreement is not inherently concerning. What is concerning, however, is that the term “emulation” which in common language implies *effort to match or surpass someone or something*. Thus, although the term does not strictly claim that a trial has been replicated, it points toward matching an RCT, and in practice an emulation can be easily read as carrying the same standing as an RCT. In the context of causal inference, this connotation is misleading. Observational analyses structured around a hypothetical trial do not recreate randomization, blinding, or even always assure external validity. They approximate certain features of a trial but remain embedded in biases arising from unavoidable limitations of routinely collected data.

Recent contributions have constructively highlighted the limitations of target trial emulation [35, 36, 37]. Yet describing the framework as an “emulation” of a target trial and subsequently enumerating its caveats leaves a residual gap between the term and what the underlying analyses can deliver. When the terminology extends into clinical guidelines, regulatory submissions, and even plain‐language summaries, terminology is often displayed without their accompanying caveats. Science demands precision of language, and any ambiguity in language can be detrimental, including in medicine when evidence is applied in patient care without required scrutiny. Thus, to complement reporting initiatives such as the TARGET guideline [37] and to more accurately and transparently reflect the difference between an ideal randomized trial and what observational data can realistically support, the framework is perhaps better termed as “approximation”. Although no term is perfect, across mathematics and statistics, “approximation” is the established term for a procedure that targets a quantity it cannot exactly reproduce, and it is consistent with language already used in causal inference. Notably, Rubin, one of the founders of modern causal inference, used the language of “approximation” when describing this very same aspiration of mimicking a hypothetical experiment with observational data given the limits of observational data [38]. “Approximation” sets a different expectation by signaling that we are getting close to a trial, not reproducing one. This difference in understood meaning of the two terms is relevant because the two terms fail in opposite directions. When “approximation” is inaccurate, it errs on the side of caution, understating how close the replication is. When “emulation” is inaccurate, it errs the other way, implying a closeness that has not been achieved and lending the analysis more weight than the evidence supports. Thus, rather than implicitly claiming trial‐like validity, investigators and readers may be prompted to consider how closely the study approximates the target trial, which components are well represented in the data, which are weakly approximated or absent, how plausible baseline comparability is given the clinical context, and how sensitive the findings are to assumptions about the data and design. Ultimately facilitating more transparent reporting, more critical appraisal of causal claims, and more scientifically rigorous interpretation of findings.

## 4 Conclusion

Target trial emulation has been a valuable framework in addressing avoidable design flaws in observational research and has improved the validity of causal inference drawn from observational studies. Yet its success has also revealed its limits. Observational studies structured as hypothetical trials remain prone to biases that are embedded in observational data. Calling the framework “approximation” may better reflect the reality of mimicking hypothetical target trial with observational data. It would preserve the methodological discipline that has made the approach influential, while recalibrating expectations toward a more honest appraisal of what available observational data can deliver.

## Author Contributions

P.V. conceived the study, searched and reviewed the literature, and drafted the manuscript.

## Funding

The author has nothing to report.

## Conflicts of Interest

The author declares no conflicts of interest.

## Data Availability

The author has nothing to report.
